# Eclipse-induced wind changes over the British Isles on the 20 March 2015

**DOI:** 10.1098/rsta.2015.0224

**Published:** 2016-09-28

**Authors:** S. L. Gray, R. G. Harrison

**Affiliations:** Department of Meteorology, University of Reading, PO Box 243, Reading RG6 6BB, UK

**Keywords:** meteorology, eclipse, measurements, nocturnal jet

## Abstract

The British Isles benefits from dense meteorological observation networks, enabling insights into the still-unresolved effects of solar eclipse events on the near-surface wind field. The near-surface effects of the solar eclipse of 20 March 2015 are derived through comparison of output from the Met Office’s operational weather forecast model (which is ignorant of the eclipse) with data from two meteorological networks: the Met Office’s land surface station (MIDAS) network and a roadside measurement network operated by Vaisala. Synoptic-evolution relative calculations reveal the cooling and increase in relative humidity almost universally attributed to eclipse events. In addition, a slackening of wind speeds by up to about 2 knots in already weak winds and backing in wind direction of about 20° under clear skies across middle England are attributed to the eclipse event. The slackening of wind speed is consistent with the previously reported boundary layer stabilization during eclipse events. Wind direction changes have previously been attributed to a large-scale ‘eclipse-induced cold-cored cyclone’, mountain slope flows, and changes in the strength of sea breezes. A new explanation is proposed here by analogy with nocturnal wind changes at sunset and shown to predict direction changes consistent with those observed.

This article is part of the themed issue ‘Atmospheric effects of solar eclipses stimulated by the 2015 UK eclipse’.

## 1. Introduction

Solar eclipse events cause a unique natural perturbation to the Earth’s atmospheric system. After the effects on solar radiation, humankind perceives solar eclipses most obviously through their effects on the near-surface atmospheric conditions: namely temperature, relative humidity (RH), and wind speed and direction. Beyond their importance as a scientific curiosity of nature, these changes can give rise to socio-economic impacts. For example, an eclipse in Svalbard led to the formation of fog that grounded air traffic [1] and the reduction of solar radiation and commonly observed reduction in near-surface wind speeds can both negatively impact the generation of renewable energy. The cooling and increases in RH attributable to eclipses are well documented. By contrast, there is less consensus on induced changes in circulation. The purpose of this study is to investigate the meteorological responses to the solar eclipse of 20 March 2015 over the British Isles with a particular emphasis on wind speed and direction. The band of totality for this eclipse was to the north of the British Isles with obscuration over the British Isles ranging from 83% (at Dover on the southeast English coast) to 98% (at Stornoway in the Scottish Outer Hebrides). Times of First contact, maximum eclipse and Fourth contact were 0825, 0932 and 1042 UTC, respectively at Dover and 0832, 0936 and 1043 UTC, respectively at Stornoway.

A review of atmospheric changes from solar eclipses which includes effects on the surface temperature and circulation is presented in [2]. Cooling of several degrees Celsius typically occurs with the magnitude dependent on the time of day, season, extent of the eclipse (if partial) and cloud conditions. The peak cooling lags the time of maximum eclipse (if a partial eclipse) or totality (if a total eclipse) by 15–30 min. Relative humidity changes are typically anti-correlated with air temperature changes and increase directly as a consequence of the cooling during eclipses. For example, a 20% increase over a plant canopy in Thiruvananthapuram, India, was observed during the 2010 annular eclipse (92% obscuration) associated with a 4°C drop in air temperature [3]. A reduction of specific humidity has also been reported and attributed to eclipse-induced subsidence of drier air [4].

A reduction in surface wind speed during eclipse events has commonly been reported and related to the characteristic stabilization of the near-surface boundary layer that occurs after sunset (e.g. [2,5–7]). By contrast, Eaton *et al.* [8] did not observe changes in wind components that exceeded natural variability, although a reduction in the variance of the wind speed was observed. Some studies have also reported sudden increases in wind speeds or gusts at the times of First and Fourth contact of the eclipse [2,9]. These may be due to downwards turbulent mixing from a jet forming above the eclipse-induced inversion (following the mechanism responsible for the formation of the nocturnal jet) [7] or atmospheric pressure changes associated with the eclipse [10]. Changes in wind direction have also been attributed to eclipse events although there is little consistency in type of change or attributed cause. H. H. Clayton was the first to propose (in 1901) that total eclipses modified the atmospheric circulation [11]. He observed a cold-cored anticyclonic circulation developed around the centre of the eclipse, extending outwards to approximately 1500 miles from the umbra. Beyond this, he observed a cyclonic circulation extending a further 1000 miles to the edge of the penumbra. Clayton attributed this cyclone to the relatively small cooling in the ‘body of the atmosphere’ [10]. The existence or otherwise of Clayton’s ‘eclipse cyclone’ was the subject of debate both at the time of the eclipse (see comment [12] and Clayton’s response [13]) and more recently. As described by Alpin *et al.* [2], some evidence consistent with the existence of of ‘eclipse cyclone’ has been described by several observational and modelling studies [14–16]. Other studies have also reported wind direction changes during eclipse events [5,6,17] but attributed them to synoptic evolution and/or local changes in mesoscale circulations such as sea breezes or mountain slope flows.

Determination of eclipse-induced meteorology changes requires a best estimate of how conditions would have evolved in the absence of the eclipse event. Estimating this evolution is a challenge; weather conditions change daily and evolve during the period of the eclipse event due to diurnal variations and synoptic-scale changes (e.g. the passage of fronts). In a previous paper [14], we diagnosed wind changes induced by the eclipse on 11 August 1999 through comparing measurements from a network of surface stations operated by the Met Office across the UK with the output of a high spatial resolution operational forecast model ignorant of the eclipse. Focusing on an inland cloud-free region (containing many measuring sites), a mean regional wind speed decrease of 0.7 m s^−1^ (1.9 knots ≡ 1 m s^−1^) and backing in wind direction (anticlockwise turning in the direction the air is going towards) of 17° during the maximum eclipse hour were diagnosed. We take the same approach here to diagnose synoptic-evolution and diurnal evolution relative eclipse changes for the 20 March 2015 eclipse. Compared with the previous study of the 1999 eclipse, vastly more data are now available; we aim here to exploit this as fully as possible. Hourly measurements from two independent surface station networks (Met Office land surface stations and a network of roadside stations used for monitoring road conditions) are compared with operational output from the Met Office weather forecast model (which did not include code to represent the eclipse). Motivated by signals in the synoptic-evolution relative changes, high temporal resolution (1-min and 20-min data) observational timeseries from selected regions are then presented to focus on the variability in these regions.

The remainder of this paper is structured as followed. An overview of the synoptic situation is given in §2. The datasets used are described in §3 beginning with observational datasets (the Met Office Integrated Data Archive System (MIDAS) land and marine surface stations dataset and Vaisala roadside dataset) and followed by the operational forecast model output. The results (§4) are presented in three subsections: §§4a and 4b present the synoptic-evolution relative eclipse-induced effects on near-surface atmospheric fields deduced from the MIDAS and roadside stations, respectively; §4c presents 1-min and 20-min temporal resolution timeseries of the observational data. Throughout §4, there is a focus on the wind fields, with temperature and RH shown primarily to demonstrate consistency between the different datasets and with previous studies on eclipse effects. Section 5 summarizes the findings and considers a new explanation for the observed wind direction changes and §6 presents some conclusions.

## 2. Synoptic overview

**Figure 1.**
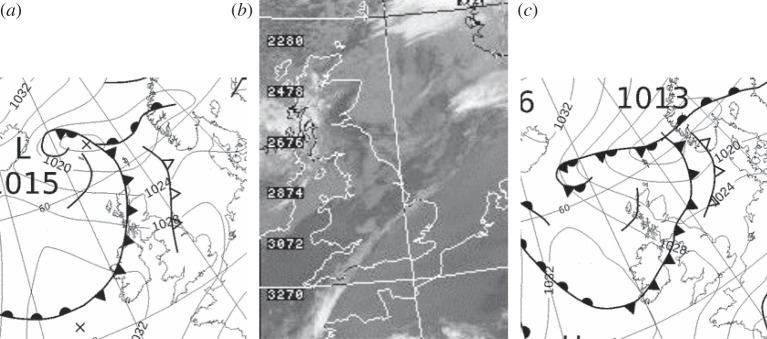
(*a*) Met Office surface analysis at 0600 UTC, (*b*) infra-red satellite image at 0922 UTC and (*c*) Met Office surface analysis at 1200 UTC. All for 20 March 2015. Surface analyses are © Crown copyright. Satellite image is courtesy of Dundee Satellite Receiving Station.

The weather situation on 20 March 2015 over the British Isles was generally cloudy with weak near-surface winds (typically less than 10 knots over England and Wales). The centre of a weak low pressure system tracked southeastwards during the morning from east of Iceland to the Scandinavian coast (figure 1*a*,*c*). The trailing cold front from this system cut across the British Isles and moved southwards during the morning. An upper-level cold front is marked in figure 1*a*,*c* to the south and east of the surface cold front at both 0600 and 1200 UTC on the surface analyses and a broad high pressure region existed to the south and west of the British Isles. The associated geostrophic surface winds can be inferred from the isobars marked on the surface analyses and were northwesterly over Scotland, Wales, Ireland and northern England and northeasterly over southern England and the Republic of Ireland at 0600 UTC but had turned more northerly by 1200 UTC. The winds were weak (inferred from the slack pressure gradients), especially to the south of the British Isles. This synoptic situation led to low-level cloud over most of the British Isles, with the exception of a clear sky band well to the south of the surface cold front (figure 1*b*); this zone provided good eclipse viewing conditions. At the time of the eclipse this band stretched across the Midlands from the county of Lincolnshire on the east coast and covered Wales and the county of Cornwall in southwest England. The calm meteorological conditions in the majority of the British Isles provided an opportunity to investigate possible eclipse-induced changes in surface weather data.

## 3. Datasets

### (a) Observations

**Figure 2.**
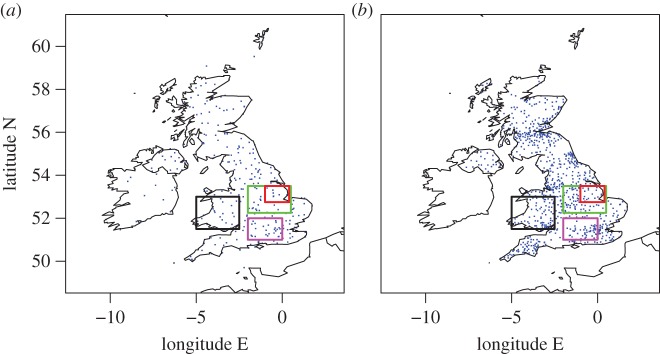
Locations of observational stations where temperature data are available at 1200 UTC on 20 March 2015 from (*a*) the MIDAS dataset and (*b*) the Vaisala roadside dataset. Magenta, black, green and red boxes in both panels mark the ‘Central southern England’, ‘Wales’, ‘Midlands1’ and ‘Midlands2’ regions, respectively, as specified in table 1.

Two observational datasets are used in this study: (i) the MIDAS surface stations dataset and (ii) measurements from a network of roadside stations operated by Vaisala for monitoring road conditions. The locations of stations from each dataset (specifically those stations with temperature observations available at 1200 UTC on 20 March 2015) are shown in figure 2. The MIDAS dataset contains land and marine surface observations from the Met Office station network across the British Isles and from other Met Office stations worldwide [18]. Hourly weather observation MIDAS data were extracted across the British Isles from the British Atmospheric Data Centre (BADC). The weather station types and message reports extracted were the same as in [14] and the same checking of quality flags was performed. Quality flags indicate the automated processing that has been performed on each measurement and whether the measurement has failed any checks. Data from 331 stations distributed across the British Isles were extracted and data were typically available (i.e. present in the station records) from approximately 280 stations for temperature and 220 stations for cloud and wind at the times analysed. Wind speed and direction (at 10 m height) and air temperature (at 1.25 m height) were analysed with data reported to 1 knot, 10 degrees and 0.1°C resolution, respectively. Wind speeds and directions are 10-min averages obtained during 20 min to 10 min prior to the reported observation hour. Wind direction observations are undefined when the wind speed is zero. It is UK practice to regard the actual observation time as 10 min prior to the observation hour; however, observations are assumed to apply to the observation hour in the analysis that follows. In addition to the hourly MIDAS data downloaded from BADC, 1-min temporal resolution data for wind speed and direction from the MIDAS stations were obtained directly from the Met Office. These data did not include quality flags.

The roadside observations were obtained courtesy of Vaisala, a company that develops, manufactures and markets products and services for environmental and industrial measurement. Vaisala operates on behalf of national agencies and local authorities a dense network of stations situated along the major UK highways. The stations vary in the types of measurements made and the frequency and timing of measurements (specifications for the Vaisala Road Weather Station RWS200 can be found in the product catalogue [19]) but hourly data for air temperature were provided from 868 stations across the UK. Data values were typically available from approximately 740, 610 and 590 stations for air temperature, wind speed and wind direction, respectively, for each time analysed. The data values do not include quality flags but come from standardized commercial products which are subject to regular calibration and maintenance to ensure validity.

Although the siting of the roadside stations is constrained by the UK road network, the majority of stations lie in relatively exposed locations away from vegetation, buildings and cuttings in order to maximize sky view factor (sky view factor is one of the main parameters affecting the behaviour of road surface temperature), reduce adverse effects on wind speed and direction, air temperature and humidity readings, and to reduce shading on the road temperature sensors particularly in low sun conditions. The aim is that the stations are sited in locations that are representative of the climatic region they represent, after allowing for power supplies and the ability to operate safely. The potential lack of representivity (for this study) arising from stations being sited according to the road network is balanced against the benefits of the very large number of stations. Almost all of the air temperature sensors are located at approximately 3.5 m above the ground with some of the wind instruments also located at this level. However, a large proportion of the wind instruments are located at the top of the roadside mast at approximately 4 m. No height corrections to the measured wind speeds are made in the data that was available. Air temperature and wind speed and direction were extracted at both hourly (whole UK) and 20-min (selected regions) intervals for this study; although greater temporal resolution data were available for some stations, the use of 20-min data provided the greatest number of stations operating at consistent temporal resolution. Temperature was reported to 0.1°C, wind speed to 0.1 m s^−1^ and direction to 1°.

### (b) Numerical model forecast

The evolution of the meteorological variables expected in the absence of the eclipse was determined using numerical model output from an operational weather forecast produced by the Met Office following the methodology of [14]. The Met Office routinely performs weather forecasts over both the whole globe and limited-area domains initialized several times each day. Different configurations of the same Met Office model, the Unified Model, are used for both weather forecasting and climate prediction. The Unified Model is an operational finite-difference model that solves the non-hydrostatic deep-atmosphere dynamical equations with a semi-implicit, semi-Lagrangian integration scheme [20]. The model includes full representation of physical processes through parametrization schemes. In the limited area configuration, the horizontal grid is rotated in latitude/longitude to yield an approximately isotropic grid measured by Euclidean distance.

The forecast used was produced by the UK variable resolution (UKV) configuration of the model [21] with an initialization time of 0300 UTC on 20 March 2015; this forecast was chosen as the latest forecast initialized before the onset of the eclipse. The UKV model configuration has a domain that extends over the British Isles and part of northern France. The grid spacing is 1.5 km in the inner domain, reducing to 4 km in a rim extending around the outer edges. There are 70 vertical levels on a staggered stretched grid extending up to approximately 70 km altitude; the lowest model levels on which temperature and horizontal wind components are held are at 5 and 2.5 m height (above the terrain), respectively. This model configuration is run operationally four times each day (initialized at 0300, 0900, 1500 and 2100 UTC using an incremental 3D-Var data assimilation scheme) out to 36 h [21]. Most model fields are output at hourly intervals including the 2-m temperature, 2-m RH and 10-m wind components used in this study. The boundary conditions for the model come from the global model forecast which runs with coarser resolution (17-km grid spacing). The operational version of the model does not include representation of eclipses in the model code and hence the forecast produced reveals how the atmospheric state would have evolved in the absence of the eclipse. The effects of model, initial condition and boundary condition errors are assumed to be small (relative to eclipse-induced effects) given the short lead time of the forecast used.

## 4. Results

### (a) Synoptic-evolution relative eclipse-induced anomalies deduced using MIDAS network observations

**Figure 3.**
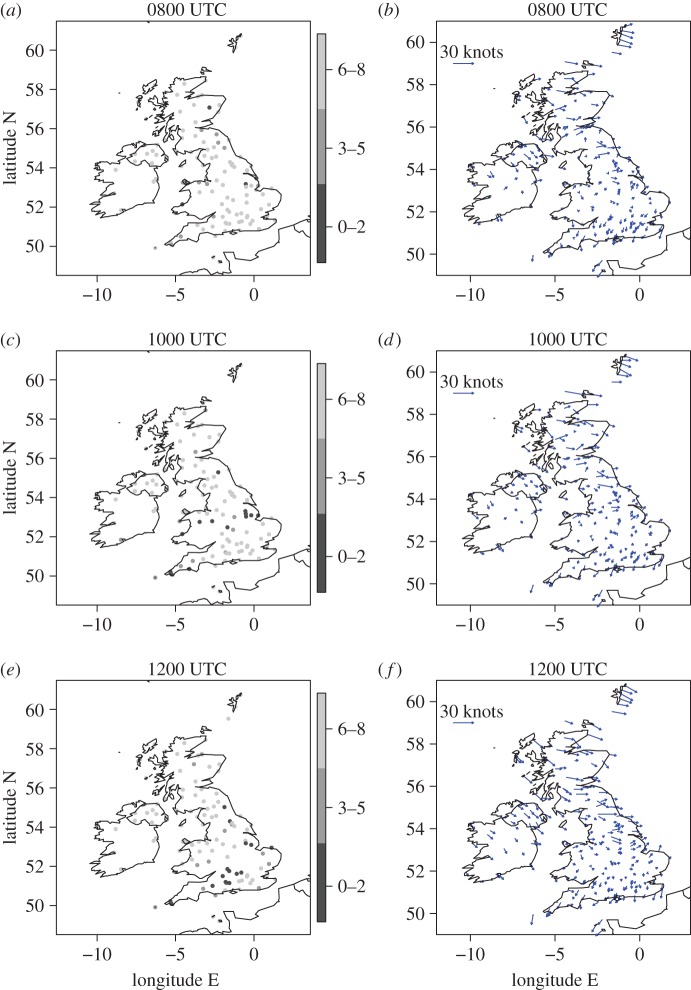
(*a,c,e*) Total cloud cover amount at MIDAS stations (oktas) and (*b,d,f*) 10-m wind vectors at MIDAS stations (with scale indicated by representative length in the upper-left of each plot) at (*a,b*) 0800, (*c,d*) 1000 and (*e,f*) 1200 UTC. Note that an okta is a unit of measurement indicating the proportion of sky covered by cloud ranging from 0 (completely clear sky) to 8 (completely overcast), with a station value of 9 indicating that the sky is totally obscured.

**Figure 4.**
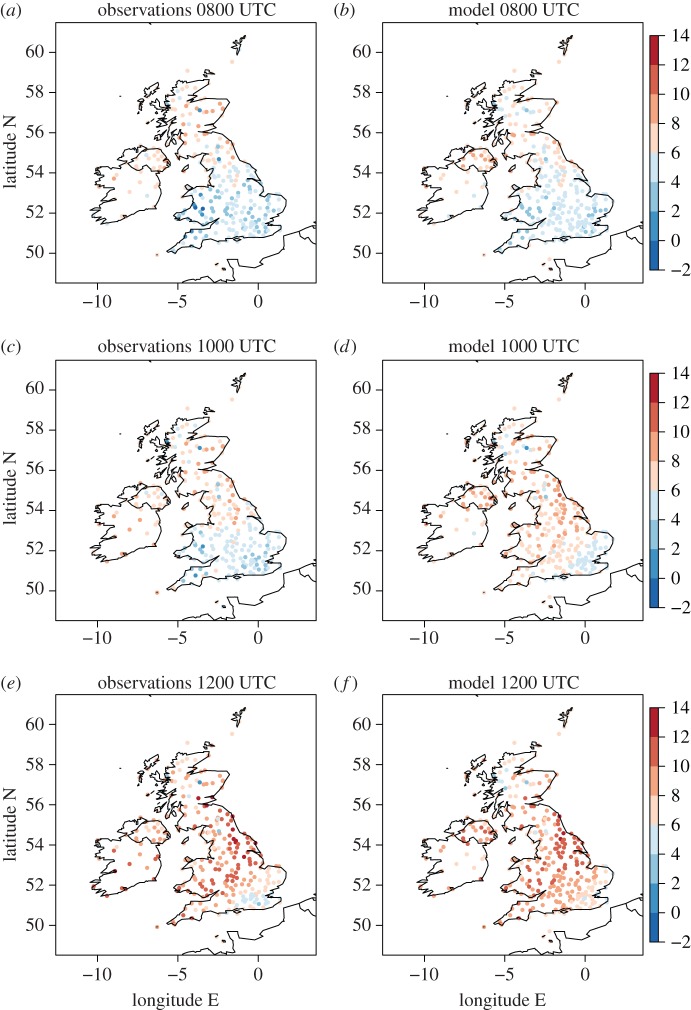
Two-metre temperature (°C) from (*a,c,e*) MIDAS stations and (*b,d,f*) model temperatures interpolated to MIDAS station locations at (*a,b*) 0800, (*c,d*) 1000 and (*e,f*) 1200 UTC.

The cloud, 10-m wind and 2-m temperature conditions before (0800 UTC), during (1000 UTC) and after (1200 UTC) the eclipse are plotted at the locations of the MIDAS stations in figure 3 and in figure 4*a,c,e*. The eclipse maximum response in atmospheric near-surface fields generally lags the maximum eclipse time (e.g. the maximum eclipse-induced temperature anomaly lags the maximum eclipse by approx. 15 min [15]). Hence, 1000 UTC is chosen to illustrate the eclipse-induced changes as this is the first available hourly data after the maximum eclipse. Data are plotted at all station points where it is available at the given times. The cloud and wind conditions (figure 3) can be compared with those inferred from the satellite imagery and surface analyses, respectively, shown in figure 1. The clear sky band across the Midlands, Wales and Cornwall at 1000 UTC is evident, with stations in this zone reporting cloud cover in the range 0–2 oktas. By 1200 UTC this band has moved slightly south and is now located in central southern England. The direction and relative strength of the 10-m wind vectors are generally consistent with the geostrophic winds inferred from the isobars in the surface analyses and are very weak in inland southern England (typical speed is less than 5 knots). At 0800 UTC temperatures to the north of the British Isles are warmer than those to the south (typically 6–8°C in the north and 4–6°C in the south, figure 4*a*, likely to be a result of less cloudy skies overnight to the south resulting in lower temperature minima there). The diurnal cycle is the dominant cause of the change in temperature during the morning (figure 4*a*–*c*) and by 1200 UTC temperatures reach up to 12–14°C in central and northeast England.

Although a comparison between figure 4*a*,*c* indicates a lack of the expected diurnal warming between 0800 and 1000 UTC, the effect of the eclipse on temperature is illustrated more directly by comparison between the observed and modelled temperatures at 1000 UTC. This is because comparison of the observed and forecast temperatures enables the changes in the observations resulting from the diurnal and synoptic-scale atmospheric evolution to be taken into account. Figure 4*b,d,f* shows the model forecast output interpolated from the 1.5 km model grid to the locations of the MIDAS stations. At 0800 UTC, prior to eclipse onset, there is a generally good agreement between the observed and forecast temperatures. At 1000 UTC temperatures across the entire British Isles are cooler than forecast and this difference is particularly apparent in the clear sky band across the midlands, Wales and Cornwall where differences reach up to approximately 4°C. By 1200 UTC observed temperatures have largely recovered to match those forecast. The exception to this is in southeast England where temperatures remain lower than forecast by approximately 2°*C*.

**Figure 5.**
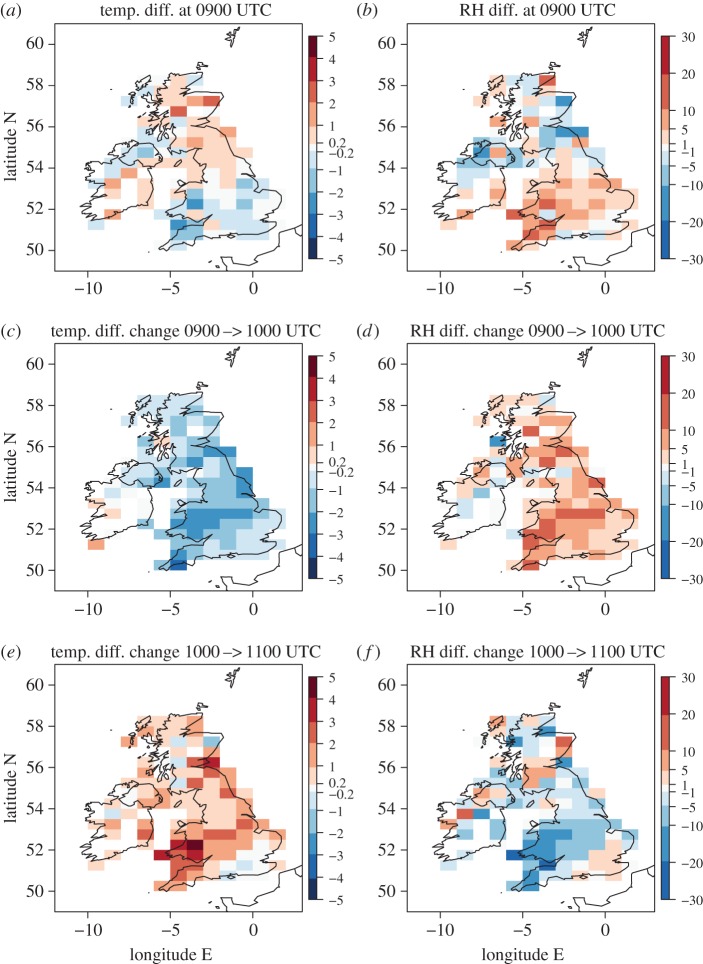
(*a,b*) MIDAS observations minus model difference for (*a*) 2-m temperature (°C) and (*b*) 2-m RH (%) at 0900 UTC. (*c,d*) Change in (observation minus model) difference between 0900 and 1000 UTC for (*c*) temperature and (*d*) RH. (*e,f*) As for (*c*,*d*) but for change in difference between 1000 and 1100 UTC. A positive value for temperature difference or change in difference indicates either warmer observed relative to modelled temperature (*a*) or a warming with time of the observed relative to the modelled temperature (*c* and *e*). A positive value for RH difference or change in difference indicates either increased observed relative to modelled RH (*b*) or an increase with time of the observed relative to the modelled RH (*d* and *f*). Station values are averaged over 1° longitude by 0.5° latitude regions. Small magnitude values are shaded light grey (−0.2 to 0.2°C; −1 to 1%).

Comparison of the 1000 UTC observed and forecast temperatures at station points (figure 4*c*,*d*) provides clear evidence of eclipse-induced cooling, greatest under clear skies. However, the observed and forecast temperatures do not exactly agree even before the onset of the eclipse, and station-to-station variability in the magnitude of the observed and forecast temperature difference exists even for neighbouring stations; these differences are expected and likely result from a combination of forecast error (even at this short forecast lead time), measurement error, and the interpolation of the model data to the station points (which does not take altitude changes into account). Figure 5*a* illustrates this difference (observed minus forecast temperature) at 0900 UTC. To allow for these differences existing before eclipse onset, figure 5*c* and *e* shows the change in the temperature difference (observed minus forecast temperature) from 0900 to 1000 UTC and from 1000 to 1100 UTC, respectively. These changes are termed ‘synoptic-evolution relative’ changes hereafter and the magnitude of these changes should be considered relative to the difference in temperature between observed and modelled temperature prior to eclipse onset. To smooth the station to station variability, the panels of figure 5 show difference fields at station points averaged over boxes of 1° longitude and 0.5° latitude (roughly square in Euclidean distance at this latitude). The time from when the synoptic-evolution relative temperature changes are determined (0900 UTC) is after the onset time of the eclipse over the British Isles. This time was chosen to enable the changes to be determined over a 1-h period focused on the time period of the likely greatest eclipse-induced changes. Considering this 1-h period will reduce uncertainty associated with the differences between the observations and forecast by providing an estimate of these differences at a time as close as possible to the time when the maximum eclipse-induced changes are expected (within the constraint of the hourly-forecast data availability). For example, the maximum temperature anomalies are expected to have occurred at approximately 0945 (approx. 15 min after the maximum eclipse at approx. 0930).

At 0900 UTC observation minus forecast differences in temperature are typically within ±1°C, with generally negative values to the south and positive values to the north of the British Isles (figure 5*a*). The north–south difference could suggest that the eclipse may have already induced a weak cooling in the clearer skies to the south (e.g. the obscuration was already approx. 45% at Reading in Berkshire at 0900 UTC), but a similar difference already exists at 0800 UTC (cf. figure 4*a*,*b*). The synoptic-evolution relative temperature change from 0900 to 1000 UTC is negative across the entire UK (isolated positive changes exist in the Republic of Ireland) implying eclipse-induced cooling occurred; typical magnitudes are 1–3°C (figure 5*b*). Many of the averaging boxes with the larger temperature changes occur in the clear sky band. Comparing the temperature change from 1000 to 1100 UTC with that from 0900 to 1000 UTC the sign is generally reversed, implying a warming in the observations relative to the forecast over this period and so a recovery from the eclipse-induced cooling. This is consistent with the improved agreement between observed and forecast temperatures an hour later at 1200 UTC (cf. figure 4*e*,*f*). The exception to this recovery is in southeast England which is associated with further (though weak) cooling from 1000 to 1100 UTC.

Changes in RH have previously been attributed to eclipse events (see §1) and the right-hand panels of figure 5 illustrate the synoptic-evolution relative RH changes in the same format as the temperature changes shown in the left-hand panels. At 0900 UTC the observations and forecast RH values generally agree within ±10% with some larger differences found over Wales, southwest England and Scotland (where local high and variable orography and coastal effects might be expected to lead to larger differences as the interpolation of the forecast data to the station points will be less representative). The synoptic-evolution relative RH changes from 0900 to 1000 UTC are generally positive, implying an eclipse-induced RH increase of the air relative to the synoptic evolution, and reach 5–20% in the clear sky band. Changes from 1000 to 1100 UTC are generally negative across England and Wales (with the exception of southeast England) implying a recovery (RH decrease) of the air towards the values that would have occurred in the absence of the eclipse. The RH changes are anticorrelated with the temperature changes implying that cooling associated with the eclipse leads to the RH increase and that this effect dominates over that due to any decrease in specific humidity. The typical magnitudes of the RH increase are also consistent with those expected from cooling at constant specific humidity: for example, a cooling of 4°C at 10°C for constant specific humidity should lead to an RH increase of approximately 20%.

**Figure 6.**
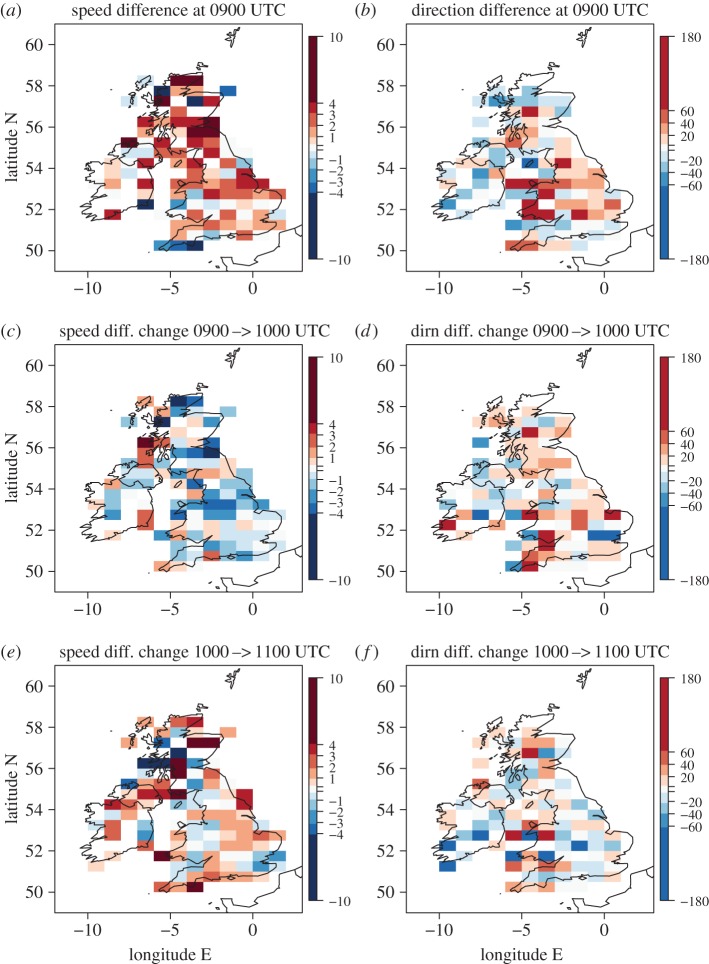
(*a,b*) MIDAS observations minus model (interpolated to station locations) difference for (*a*) 10-m speed (knots) and (*b*) 10-m direction (°) at 0900 UTC. (*c,d*) Change in (observation minus model) difference between 0900 and 1000 UTC for (*c*) speed and (*d*) direction. (*e,f*) As for (*c,d*) but for change in difference between 1000 and 1100 UTC. A positive value for speed difference or change in difference indicates either stronger observed relative to modelled wind (*a*) or a strengthening with time of the observed relative to the modelled wind (*c* and *e*). A positive value for direction difference or change in difference indicates either a backed observed relative to modelled wind (*b*) or a backing with time of the observed relative to the modelled wind (*d* and *e*). Station values are averaged over 1° longitude by 0.5° latitude regions. Small magnitude values are shaded light grey (−0.2 to 0.2 knots; −4 to 4°). Note that in panel (*a*) there is one data value of −14 knots; this is shaded in the colour indicted as the −10 to −4 knot range.

Eclipse-induced effects on temperature and RH are well understood and documented. By contrast, the effects on wind speed and direction are more controversial (see §1). Figure 6 shows the synoptic-evolution relative changes in wind speed and direction in the same format as in figure 5. At 0900 UTC observed wind speeds and directions are generally within ±2 knots and ±40° of the forecast values over southern England, but larger differences exist elsewhere, especially over Scotland for wind speed and Wales for wind direction. As with the RH field, larger differences over high and variable orography are not surprising; note also that the stations are less densely located over Scotland and Wales than in England (figure 3*b*,*d*,*f*) reducing the smoothing effect of the box averaging. Focusing on England, a generally negative synoptic-evolution relative change in wind speed occurs from 0900 to 1000 UTC of up to 4 knots followed by further changes of typically ±2 knots from 1000 to 1100 UTC with increases slightly dominating over decreases. These changes imply a reasonably consistent eclipse-induced weakening of the winds followed by some signs of a recovery. The changes are less consistent across the British Isles than the equivalent changes in temperature and RH. This can partly be attributed to the very weak winds over England at this time (figure 3*b*,*d*,*f* and note that cup anemometers also have a starting wind speed, i.e. a minimum wind speed that can be recorded, which is typically approx. 1–4 knots [22]). Synoptic-evolution relative changes in direction are also not very consistent across the British Isles (figure 6*d*,*f*). However, there is a generally positive synoptic-evolution relative wind direction change across England from 0900 to 1000 UTC followed by a generally negative change from 1000 to 1100 UTC. In the calculation, a positive change means that the angle anticlockwise relative to East that the wind vector points towards in the observations has increased relative to that angle in the forecast and so a backing of the wind direction; a negative change implies the observed wind direction has veered relative to the forecast. Hence, we conclude that the synoptic-evolution relative wind evolution reveals a reasonably coherent weakening in the (already weak) wind speeds and the suggestion of a backing of wind direction over England during the eclipse.

The lack of strongly robust eclipse-induced wind response motivates a further analysis using the independent roadside station network, which has an enhanced density of measurement sites.

### (b) Synoptic-evolution relative eclipse-induced anomalies deduced using Vaisala roadside network observations

**Figure 7.**
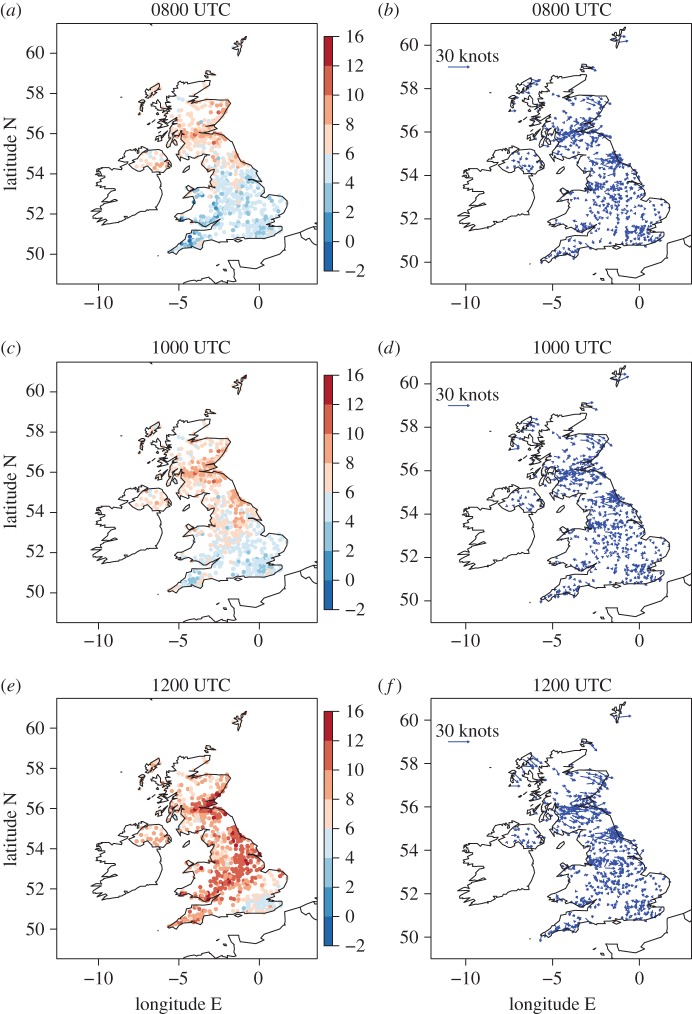
(*a,c,e*) temperature at roadside stations and (*b,d,f*) wind vectors at roadside stations at (*a,b*) 0800, (*c,d*) 1000 and (*e,f*) 1200 UTC.

The greater density of the roadside network stations compared to the MIDAS stations used and general consistency of the temperature and winds measured there with those from the MIDAS station network (and model forecast prior to eclipse onset) can be seen by comparison of figure 7*b,d,f* with figure 3 (right panels) and figure 4. (Note that roadside observations were not available for the Republic of Ireland.) The roadside station temperatures are very consistent with the MIDAS station observations and forecast generally the same temperatures (in the same regions) within the 2°C discretization used for plotting. The warmest roadside station temperatures slightly exceed those from the MIDAS stations which accounts for the warmest colour band covering 4°C in figure 7*a,c,e* instead of 2°C as in figure 4. The wind speeds measured by the roadside stations are generally weaker than those measured by the MIDAS stations. This is likely to be attributable to the lack of numerical adjustment of the height of wind measurements from the measurement height to 10 m (as is performed where required for the MIDAS observations). The wind directions across the UK from the roadside and MIDAS observations are regionally consistent.

**Figure 8.**
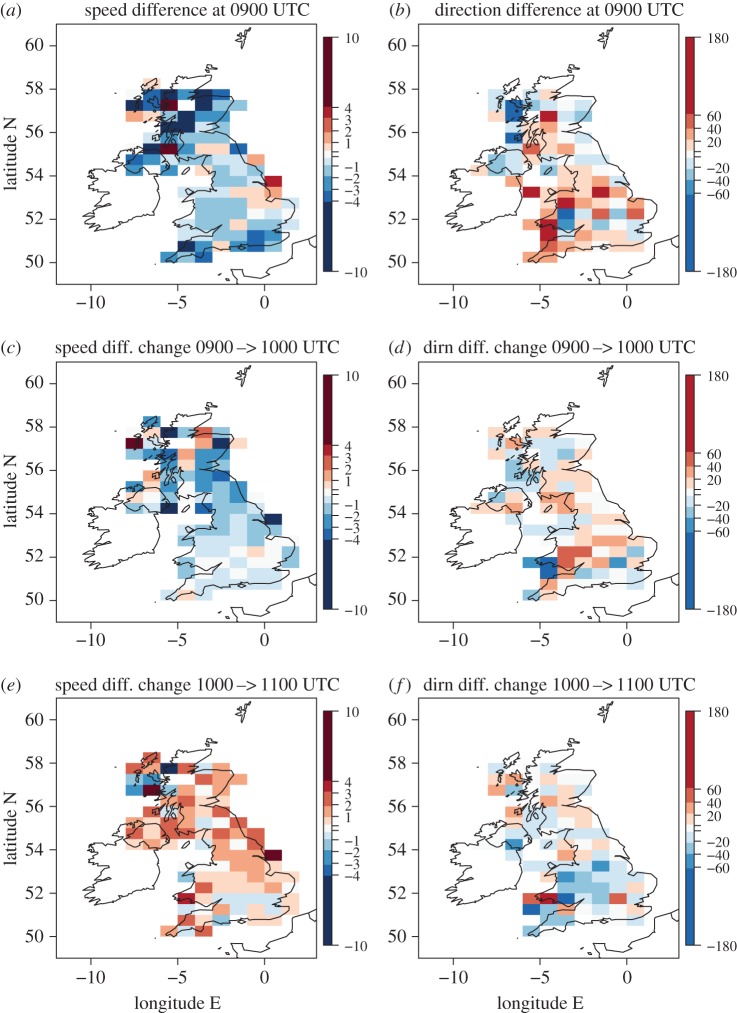
As for figure 6, but for comparison of the roadside observations with the model. All data values are within ranges indicated on the colour bars.

Figure 8 shows the synoptic-evolution relative changes in wind speed and direction for the roadside observations; this is derived in the same way as for the MIDAS observations shown in figure 6. Overall, the fields plotted appear more spatially coherent for the roadside observations than for MIDAS observations, especially over England and Wales; this is likely a consequence of the greater density of stations for the roadside observations. At 0900 UTC, the observed wind speeds can be seen to be generally weaker than the forecast winds by 1–2 knots over Wales and inland England areas though by more over Scotland and along the southern UK coast where winds were stronger (figure 8*a*). This may partly be attributable to the eclipse since 0900 UTC is after eclipse onset (although the observed MIDAS wind speeds were generally stronger than the forecast ones at 0900 UTC (figure 6*a*)); other likely reasons for this are discussed above based on comparison of the roadside and MIDAS wind vectors. Observed roadside wind directions are generally within ±20° of those forecast (figure 8*b*) with a slight preference for the observed winds to be backed relative to those forecast, as also found with the MIDAS observations (figure 6*b*). The synoptic-evolution relative changes in roadside wind speed and direction from 0900 to 1000 UTC and from 1000 to 1100 UTC are consistent in sign with those found for the MIDAS observations, especially over England, and the greater spatial coherence of the patterns lends confidence that they are robust.

These robust synoptic-evolution relative changes now motivate the closer examination of high temporal resolution (1-min and 20-min data) observations from selected stations.

### (c) Regional eclipse-induced surface meteorological change deduced from high temporal resolution observations

**Figure 9.**
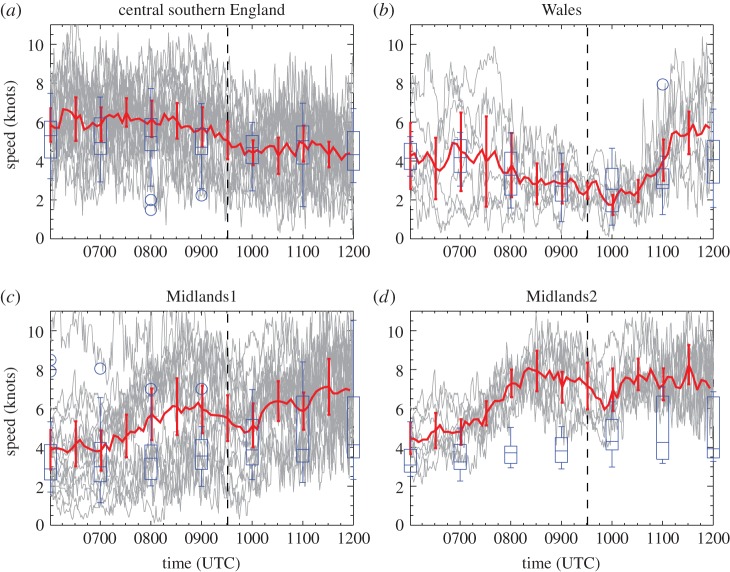
One-minute temporal resolution MIDAS 10-m wind speed data (knots) as a function of time for sets of stations in four regions: (*a*) central southern England, (*b*) Wales, (*c*) Midlands1 and (*d*) Midlands2. Stations are selected within the latitude and longitude limits at which 1-min wind speed data are available at all times and the speed is non-zero at all times. Vertical dashed line marks the approximate time of the maximum partial eclipse (0930 UTC). Thick red line shows the mean value with error bars given by ±1.96 s.d. (yielding the 95% CI). Model forecast values interpolated to station locations are overplotted in blue in box and whiskers format. The box encloses the interquartile range with the horizontal line marking the median value. The whiskers extend out to the maximum or minimum value of the data, or to 1.5 times either the 1st or 3rd quartile if there is data beyond this range. Outliers are identified with circles. Number of stations plotted is (*a*) 17, (*b*) 7, (*c*) 16 and (*d*) 8. The upper bound of speeds plotted is limited for clarity so data from individual stations occasionally exceeds this.

**Figure 10.**
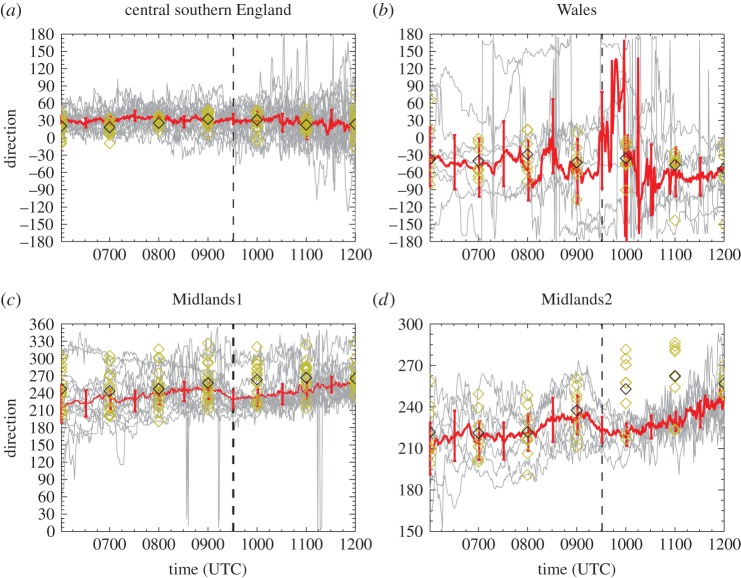
As figure 9, but for wind direction. Wind directions are described conventionally, as the angle clockwise from North that the wind is coming from (e.g. an angle of 90° is an easterly wind). Stations are selected within the latitude and longitude limits at which 1-min wind speed data and direction data are available at all times and the speed is non-zero at all times. Model forecast values interpolated to station locations are overplotted in orange diamonds (rather than as box and whiskers as in figure 9); black diamond denotes mean values. Number of stations plotted is the same as in figure 9. Note *y*-axis varies between panels.

**Figure 11.**
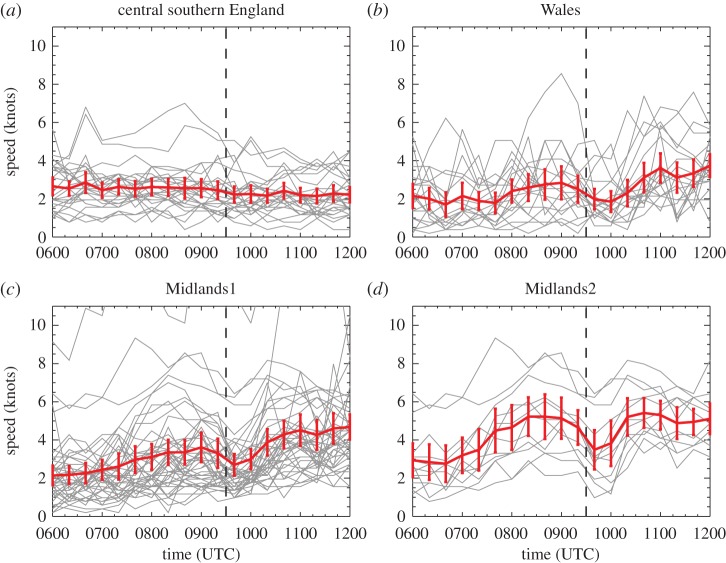
As figure 9, but for 20-min temporal resolution roadside wind speed data. Number of stations plotted is (*a*) 26, (*b*) 19, (*c*) 44 and (*d*) 12.

**Figure 12.**
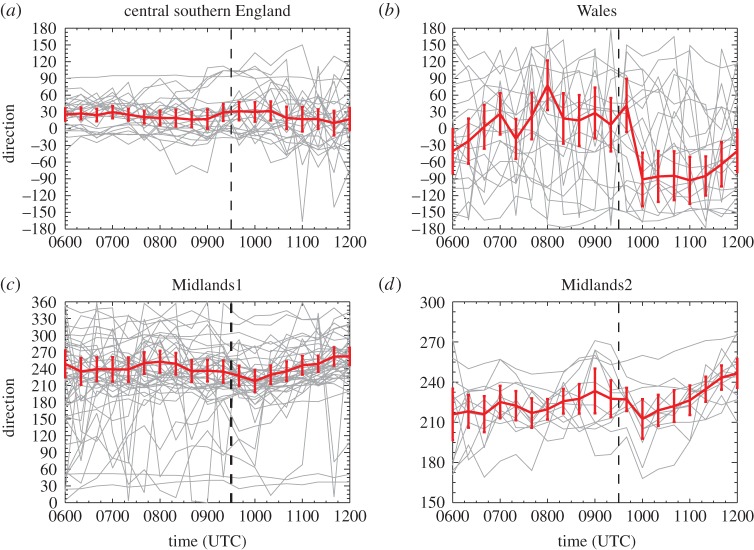
As figure 11, but for wind direction. Number of stations plotted is the same as in figure 11.

**Figure 13.**
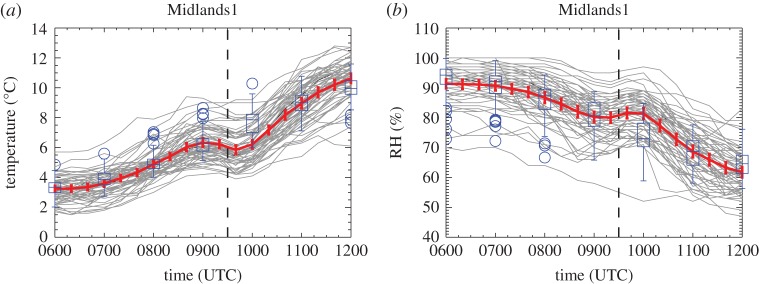
Twenty-minute temporal resolution roadside observations as a function of time for (*a*) temperature and (*b*) RH in the Midlands1 region only. Stations are selected within the latitude and longitude limits at which 20-min (*a*) temperature and (*b*) RH data are available at all times. Vertical dashed line marks the approximate time of the maximum partial eclipse (0930 UTC). Thick red line shows the mean value with error bars given by ±1.96 s.d. (yielding the 95% CI). Model forecast values interpolated to station locations are overplotted in blue in box and whiskers format (as in figure 9). Number of stations plotted is 62 (for both fields).

The advantage of analysing 1-min and 20-min temporal resolution station data is that eclipse-induced changes occurring on timescales of less than an hour can be resolved. The disadvantages are that synoptic-evolution relative changes cannot be easily determined for comparison (the operational model output is only available on the hour for fields analysed here) and the 1-min MIDAS wind data are very noisy compared with the hourly MIDAS wind data (which is provided as an average over a 10-min period). Figures 9–12 show wind speed and direction data at 1-min (MIDAS observations) and 20-min (roadside observations) intervals for four selected regions; these regions are marked on figure 2 and the specifications are given in table 1. The selection of these regions was motivated by the robust eclipse-induced changes in wind speed and direction (relative to the synoptic evolution) found over England in §4a,b and the preferential occurrence of large eclipse-induced changes in temperature and RH in the clear sky band across the midlands, Wales and Cornwall. The Wales and Midlands1 regions were selected as covering the cloud free regions over central England at 1000 UTC. Midlands2 is a subregion of Midlands1, chosen to focus on the clearest region and such that there is consistency in the wind speed station data for the stations chosen (i.e. the evolutions of wind speed and direction follow tight plumes). The central southern England region was selected as a cloudy inland region just south of the cloud-free band with lots of stations. Figures 9–13 show the data from individual stations (thin grey lines) and mean values (bold red lines) with error bars. UKV model forecast output interpolated to the station locations is also shown in the panels showing the 1-min MIDAS data (figures 9 and 10) and the temperature and RH values for the 20-min roadside data (figure 13). In figures 9 and 13, the model output data are shown in box and whisker format; in figure 10, the orange diamonds mark the model values at the stations and black diamonds mark the mean of the station values. Stations within the regions that recorded zero wind speed at any time within the limits plotted were excluded from the analysis so as not to bias the mean values of wind speed and wind direction (direction is undefined when speed is zero). This constraint removed several stations from analysis, particularly in the Wales region where the winds were weakest.

**Table 1.** Specification of regions. The location of the regions is shown in figure 2.

region	latitude limits (°N)	longitude limits (°E)	comment
central southern England	51, 52	−2, 0	cloudy, inland
Wales	51.5, 53	−5, −2.5	clear band
Midlands1	52.25, 53.5	−2, 0.5	clear band
Midlands2	52.75, 53.5	−1, 0.4	clear band; subset of Midlands1

Beginning with the 1-min MIDAS observations, figure 9 shows that wind speeds evolved due to synoptic evolution and the diurnal cycle during the time period shown (gradually decreasing on average over the central southern England region, for example). However, within this evolution, over a several hour timescale, a more rapid evolution can be discerned around the time of the eclipse (the approximate time of the maximum partial eclipse, 0930 UTC, is marked by the bold dashed line). A reduction in wind speed from approximately 0900 to 1000 UTC is clearly evident in the central southern England and Midlands regions (in the central southern England region this the post-eclipse increase is somewhat obscured by the general reduction in wind speed during the period shown). Over Wales, a reduction in the range of wind speed during the eclipse is more apparent than a reduction in the mean wind speed. The reduction in wind speed of about 2 knots is followed by a rapid recovery in the Midlands1 and Midlands2 regions over approximately 15 min. By contrast, the wind speeds forecast by the UKV model do not undergo a rapid reduction around the time of the eclipse. The wind speeds are under-predicted by the forecast in the central southern England, Midlands1 and Midlands2 regions prior to the eclipse and so are a better match to those observed after the eclipse. In the Wales region, the mean forecast wind speed is a good match to that observed throughout the period plotted; however, a reduction in the spread across stations is not observed in the forecast wind speeds, unlike in the observations. While the effect of other forcings on the wind speed cannot be ruled out, the change observed around the time of the eclipse is attributed here to the eclipse due to its rapidity, the lack of similar wind speed changes in the model forecast winds, and the consistency of the wind speed changes with those observed in other eclipse events.

In figure 10, a backing from approximately 0900 to 0930 UTC by approximately 20°, followed by a slow veering, in the wind direction during the eclipse is clearly evident in the Midlands1 and Midlands2 regions. There is no obvious wind direction change associated with the eclipse over the cloudy central southern England region. Over Wales the wind directions are very variable, as expected given the hills and mountains in this region which can dominate local wind flows; it is not possible to discern an eclipse-induced wind direction change in this region. The forecast wind directions are a good match to those observed over the central southern England and Wales regions (although the observed range of directions is enhanced after the eclipse relative to the model forecast). In the Midlands1 and Midlands2 regions, the forecast and observed wind directions are a good match until 0900 UTC after when the forecast winds continue to veer whereas the observed winds back leading to a difference of approximately 30° at 1000 UTC. The timescale of the wind speed and direction changes attributable to the eclipse is approximately 1 h which provides rationale for the calculations of synoptic-evolution relative changes over 1-h periods presented in figures 5, 6 and 8.

The 20-min roadside observations have similar temporal evolutions to the 1-min MIDAS observations. The wind speeds are generally weaker in the roadside observations (e.g. cf. figure 11*a* with 9*a*) which is consistent with the systematic bias seen across the entire UK and discussed in §4b. A reduction in mean wind speed of about 2 knots from approximately 0900 to 1000 UTC is evident in the Wales, Midlands1 and Midlands2 regions but not discernable in the central southern England region (figure 11*a*). The roadside observation wind direction timeseries for the Midlands1 and Midlands2 regions show the same pattern of backing followed by veering during the eclipse period as the equivalent MIDAS observation plots (cf. figures 12*c*,*d* and 10*c*,*d*) although the wind directions are more widely spread within the each region. In the central southern England region (figure 12*a*), the mean direction veers during the eclipse period relative to the direction before and after the eclipse although this does not seem to be directly attributable to the eclipse as the veering takes place transiently during the 20 min after 0900 UTC, and is not sustained throughout the eclipse. In the Wales region (figure 12*b*), the mean wind backs sharply at 0940 UTC. The large variability in the wind directions in this region together with the sharpness of the backing suggest that this change also cannot be attributed to the eclipse.

Confidence in the wind speed reduction and backing attributed to the eclipse can be qualitatively assessed from the size of the changes during the eclipse compared with the size of the error bars plotted in figures 9–12 (the error bars indicate the 95% confidence interval in the mean). The error bars are generally statistically smaller for the roadside observations than for the MIDAS observations due to the larger number of available stations. The highest confidence in the eclipse-induced changes is found by considering the Midlands1 region, the larger of the two Midlands regions located in clear skies, and the roadside data. The size of the eclipse-induced anomalies can only be approximated from the plots since the effects of the synoptic and diurnal evolutions cannot be precisely quantified. The estimated wind speed reduction of about 2 knots exceeds the confidence interval of 1.3 knots calculated at 0930 (figure 9*c*). However, the estimated backing of approximately 20° is about half the confidence interval of 38° and hence cannot be considered robust. Confidence in the changes in these wind fields can be contrasted with that in temperature and RH. Figure 13 shows the temporal evolution of temperature and RH from the roadside observations in the Midlands1 region. Both fields clearly fail to completely recover from the eclipse-induced changes leading to a lag in the morning warming and RH reduction of the near-surface air. Despite this complication, the eclipse-induced changes of perhaps 1°*C* and 10% clearly exceed the confidence intervals (calculated as 0.6°C and 3.6% at 0930).

## 5. Discussion

An analysis has been presented of the near-surface atmospheric anomalies over the British Isles attributable to the partial solar eclipse of 20 March 2015. Previous research has demonstrated consistent cooling and associated RH increase associated with eclipse events. However, the wind field response has been more unclear with some researchers identifying a response and others not. To account for the synoptic-scale evolution during the eclipse, eclipse-induced anomalies have been determined through comparison of meteorological observations against the output of a state-of-the-art high-resolution operational weather forecast over the UK; the solar eclipse was not represented in the model code and so the forecast provides a ‘best guess’ of the forecast evolution that would have occurred in the absence of the eclipse. Two types of meteorological measurements were analysed: (i) measurements from the MIDAS Land Surface Stations (both hourly observations over the British Isles and 1-min observations from selected stations) and (ii) measurements from a roadside measurement network operated by Vaisala to monitor road conditions. The roadside network is extensive over the UK with 868 stations used compared with the 331 MIDAS stations from which hourly weather observations were obtained. Whereas the MIDAS dataset is routinely used for British Isles meteorological analysis, we believe this is the first meteorological analysis using the unique roadside network dataset.

Weather conditions for observing eclipse-induced surface weather changes were poor over most of the British Isles with widespread cloud and relatively weak winds. However, a band of clear skies extended across middle England, Wales and into northern Cornwall. Hence, in addition to the British Isles-wide analysis presented here, 1-min and 20-min observations have been presented from regions within this clear sky band and from a region with contrasting weather conditions: cloudy central southern England. MIDAS wind and cloud observations over the British Isles are consistent with the Met Office surface analyses (in terms of direction and relative strength as inferred from the isobars) and satellite imagery. MIDAS temperature observations show show the expected diurnal warming during the morning and comparison with the model output illustrates the cooling attributable to the eclipse (reaching up to 4°C at station points in the cloud-free regions).

Synoptic-evolution relative eclipse-induced gridded (1° longitude by 0.5° latitude grid box averages) temperature anomalies and RH anomalies (inferred from comparing the changes in the MIDAS observations with those from an operational model forecast ignorant of the eclipse) reach up to 3°C and 20%, respectively, in the cloud-free band. Synoptic-evolution relative eclipse-induced gridded wind speed and direction changes are less homogeneous across the UK than for temperature and RH but, focusing on the clear sky band, there is reasonably consistent weakening (by up to 2 knots in the already weak winds) and backing in the wind direction (by 20–40°) during the eclipse followed by a somewhat consistent strengthening and veering after the eclipse.

The temperature and wind evolution during the eclipse from the roadside observations are consistent with those in the MIDAS observations although the winds are generally weaker. This difference is likely attributable to a lack of numerical adjustment of the height of wind measurements from the measurement height to 10 m (as is performed where required for the MIDAS observations); sheltering of the roadside stations may also contribute. The larger number of roadside stations is highly advantageous as it yields smoother gridded wind speed and direction distributions over the UK compared with the MIDAS dataset. A consistent weakening and backing followed by strengthening and veering compared to the model output is found fairly consistently across the clear sky regions in middle England and Cornwall.

High temporal resolution (1-min and 20-min data) observations for stations within three clear sky regions and one cloudy region illustrate the timescales during which the eclipse-induced wind changes occur. Wind speeds slacken from shortly before 0900 UTC to nearly 1000 UTC by up to about 2 knots in all regions at the MIDAS stations and three of the four regions at the roadside stations (the exception being the cloudy central southern England region). Backing of approximately 20° occurs over the same timescale although this is only evident in the clear sky Midlands regions (at both station types); no clear directional changes are observed in the cloudy central southern England region and the directional changes are highly variable in the Wales region (hypothesized to arise due to local slope-induced flows in this orographic region). The sign and magnitude of these regional changes are consistent with those determined from MIDAS observations from the 11 August 1999 eclipse over the UK (0.7 m s^−1^ and 17°) [14]; this eclipse was at its maximum over the UK (total over southwest UK) at about 1015 UTC, so at a similar time of day to the 20 March 2015 event. This consistent wind response during two different eclipse events and determined using two independent observational networks provides confidence that wind direction changes occur robustly during eclipse events over inland regions with low orography.

The backing of the wind direction observed during the eclipse is reminiscent of the changes in wind direction which can also occur at sunset leading, in some circumstances, to a jet—the nocturnal jet—of relatively enhanced wind speeds above the surface [23,24]. The situation leading to a nocturnal jet was originally studied by Blackadar [25]; Thorpe & Guymer [26] used a layer model to consider the effect of surface friction on the jet and showed that the jet maintained its increased speed through decoupling of the jet from the surface. This decoupling minimizes the surface drag on the flow, a further consequence of which is a change in wind direction. Wind direction changes can also occur without the full decoupling of the flow associated with the nocturnal jet if the relative effects of surface drag, the pressure gradient force and Coriolis acceleration change. A change in the boundary layer depth can lead to such wind direction changes. During a typical convective cloudy day, a convective boundary layer steadily develops which then transitions to a shallow stable layer after sunset (e.g. [27]). The circumstances of a substantial eclipse are similar, but the changes occur more rapidly. Wind profiles of the whole boundary layer were obtained using an Ultra High Frequency (UHF) band Doppler Radar during the 11 August 1999 eclipse by Girard-Ardhuin *et al*. [28], who reported appreciably lessened reflection from the boundary layer top, associated with a suppression of turbulence and boundary layer reduction. A decline in the boundary layer height was also directly observed during the 29 March 2006 solar eclipse in Greece by [29] who showed, using lidar and balloon soundings in clear conditions over Athens, that mixed layer heights during the 84% partial eclipse were reduced from 800 m to 620 m.

For the 20 March 2015 eclipse, there is observational evidence of the depth of the boundary layer available from a radiosonde ascent launched from Reading (51.44° N, 0.94° *W*) before eclipse onset (0848 UTC) which landed in Winchester (31 miles southwest) at 1013 UTC, and from a UHF Doppler radar wind profiler operating at Cardington (52.10° *N*, 0.42° W, [30]) throughout the eclipse event (neither sets of data are shown). The boundary depth, inferred from the height of the temperature inversion, decreases during the morning in the vicinity of Reading, but this decrease is not inconsistent with the synoptic evolution expected from model profiles. By contrast, at Cardington there is evidence from the wind profiler of a slow reduction in boundary layer depth throughout the morning (from approx. 900 m at 0300 UTC to approx. 600 m at 0800 UTC and then to 500 m at 1000 UTC) before the boundary layer deepens again after noon. Thus, the boundary layer is shallowest at about the time of the maximum eclipse. While this boundary depth reduction cannot be confidently attributed to the eclipse, a similar decrease in boundary layer depth is not observed in hourly model profiles (again from a model ignorant of the eclipse) taken at Cardington. Burt [31] provides other evidence of boundary layer changes during this eclipse through observations showing a reduction in cloud base height of 20 m during the passage of the eclipse, together with other boundary layer changes in temperature and wind speed consistent with findings from the wider observation network analysed in this paper. Comparison of the operational weather forecasts made by the Met Office UKV model against a forecast made by the same model but including a parametrization for the eclipse in the model code shows shallower boundary layer depths in the clear sky region, and in Scotland and Northern Ireland, in the forecast from the model with the eclipse parametrization at the time of the maximum eclipse [32]. These model results reveal that a boundary layer depth change attributable to the eclipse would be expected to have occurred at Cardington but not at Reading, consistent with the available observations described above.

Effects of boundary layer depth changes can be represented by a theoretical model of the boundary layer such as that of [26]. This model relates the surface drag coefficient, *C*_D_, and boundary height, *h*, with the northwards, *u* and westwards, *v*, components of the wind speed by two coupled equations:

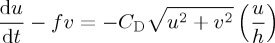
and

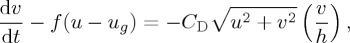
where *f* is the Coriolis parameter and *u*_*g*_ is the free geostrophic wind speed above the layer. These coupled time-dependent ordinary differential equations can be solved numerically for *u* and *v* as a function of time *t*, if representative values of *C*_D_ and *h* are assumed, given initial conditions for *u* and *v* at *t*=0.

**Figure 14.**
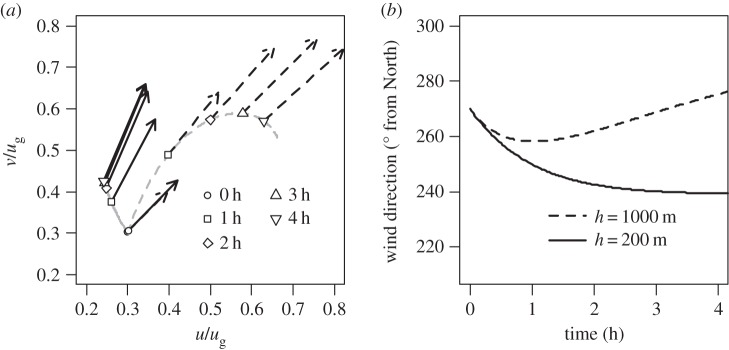
Illustrative calculations of the variation in wind direction for atmospheric boundary layer heights of 1000 m (dashed lines) and 200 m (solid lines), using the theoretical model in [26]. (*a*) Hodograph showing the wind vectors at hourly intervals, beginning from initial wind vector components *u* and *v* of 3 ms^−1^ (at *t*=0). The values of *u* and *v* have been normalized by an assumed geostrophic wind speed *u*_g_ of 10 ms^−1^. (*b*) Time series of the calculated wind directions for the two boundary layer heights. (Drag coefficient *C*_D_=8×10^−3^, and Coriolis parameter calculated for 50°N.)

Figure 14*a* shows time-dependent numerical solutions (i.e. the variations of *u* and *v* with time *t*) calculated for two different layer depths, beginning with initial values of *u* and *v* (at *t*=0) chosen to approximate the wind speeds in the Midlands regions around the eclipse time. (A fourth order adaptive Runge–Kutta numerical method was used for these calculations, from Press *et al.* [33].) In figure 14*a,* the magnitudes of the wind vectors at hourly intervals found by integration from the initial values are plotted against each other in hodograph form, to show the associated changes in direction with time. The two boundary layer heights were selected to give an illustrative comparison between the situations of sustaining an established boundary layer in clear skies, and the much shallower boundary layer which may be formed during an eclipse. With time, the wind direction in the shallow boundary layer case turns anticlockwise, compared with the more constant wind direction maintained in the deeper boundary layer case. Figure 14*b* shows the continuous variation in wind directions against time (from which the sampled hourly values of figure 14*a* were obtained.) Although the exact surface and flow parameters of the regions are not attempted to be represented, the wind direction change apparent under these assumptions is not inconsistent with that observed in figure 12*c*,*d*. Similar nocturnal oscillatory behaviour has been observed in near surface winds from the Cabauw observatory [23].

## 6. Conclusion

In addition to the well-documented cooling, increase in RH and slackening of the near-surface wind speed, wind direction changes can also be attributed to an eclipse. While confidence in wind speed changes is similar to that in temperature and RH, these far exceed confidence in the wind direction changes. Nevertheless, the wind direction changes remain of broader interest, because of their relationship to the supposed ‘eclipse wind’. It is noteworthy that the wind direction changes have been found to be robust across the two eclipse events in 1999 and 2015, with the latter analysis here using two independent observation networks. Some theoretical support for the wind direction changes identified in the clear sky inland regions is provided by considering the rapid eclipse-induced cooling as analogous to the formation of nocturnal wind structures. This may provide a more parsimonious explanation for the surface wind direction changes observed than the substantial mesoscale structure postulated by Clayton [11].

Finally, a spatially dense, high temporal resolution (subhourly) meteorological observation network is invaluable for studies of meteorological responses to transient phenomena. The roadside network here employed now provides a further novel valuable source of such meteorological measurements.

## Data accessibility

The hourly weather observations MIDAS dataset and operational output from the MetUM is available through the British Atmospheric Data Centre (BADC). The data files for the Vaisala roadside network and 1-min Met Office surface station wind measurements were obtained directly from Vaisala and the Met Office, respectively.

## References

[1] Sjöblom A 2010 A solar eclipse seen from the High Arctic during the period of midnight sun: effects on the local meteorology. *Meteorol. Atmos. Phys.* 107, 123–136. (10.1007/s00703-010-0070-3)

[2] Aplin KL, Scott CJ, Gray SL 2016 Atmospheric changes from solar eclipses. *Phil. Trans. R. Soc. A* 374, 20150217 (10.1098/rsta.2015.0217) 27550760

[3] Murthy BS, Latha R, Sreeja P, Dharmaraj T 2012 Transient land breeze: Eclipse induced wind flow modifications—observations over plant canopy. *J. Atmos. Sol.-Terr.* 89, 33–39. (10.1016/j.jastp.2012.07.010)

[4] Bhat GS, Jagannathan R 2012 Moisture depletion in the surface layer in response to an annular solar eclipse. *J. Atmos. Sol.-Terr.* 80, 60–67. (10.1016/j.jastp.2012.02.025)

[5] Namboodiri KVS, Dileep PK, Mammen K, Ramkumar G, KiranKumar NVP, Sreenivasan S, Kumar BS, Manchanda RK 2011 Effects of annular solar eclipse of 15 January 2010 on meteorological parameters in the 0 to 65 km region over Thumba, India. *Meteorol. Z.* 20, 635–647. (10.1127/0941-2948/2011/0253)

[6] Founda D, Melas D, Lykoudis S, Lisaridis I, Gerasopoulos E, Kouvarakis G, Petrakis M, Zerefos C 2007 The effect of the total solar eclipse of 29 March 2006 on meteorological variables in Greece. *Atmos. Chem. Phys.* 7, 5543–5553. (10.5194/acp-7-5543-2007)

[7] Winkler P, Kaminski U, Köhler U, Riedl J, Schroers H, Anwender D 2001 Development of meteorological parameters and total ozone during the total solar eclipse of August 11, 1999. *Meteorol. Z.* 10, 193–199. (10.1127/0941-2948/2001/0010-0193)

[8] Eaton D, Hines JR, Hatch WH, Cionco RM, Byers J, Garvey D, Miller DR 1997 Solar eclipse effects observed in the planetary boundary layer over a desert. *Bound. Layer Meteorol.* 83, 331–346. (10.1023/A:1000219210055)

[9] Nymphas EF, Adeniyi MO, Ayoola MA, Oladiran EO 2009 Micrometeorological measurements in Nigeria during the total solar eclipse of 29 March. *J. Atmos. Sol.-Terr.* 71, 1245–1253. (doi:10.1016./j.jastp.2009.04.014)

[10] Clayton HH 1908 The meteorology of total solar eclipses, including the eclipse of 1905. *Ann. Harv. Coll. Obs.* 58, 192–216.

[11] Clayton HH 1901 The eclipse cyclone and the diurnal cyclones. *Ann. Astron. Obs. Harv. Coll.* 43, 5–22.

[12] Bigelow FH 1901 Clayton’s eclipse cyclone and the diurnal cyclones. *Science* 13, 589–591. (10.1126/science.13.328.589) 17752131

[13] Clayton HH 1901 Clayton’s eclipse cyclone and the diurnal cyclones. *Science* 13, 747–750. (10.1126/science.13.332.747) 17830164

[14] Gray SL, Harrison RG 2012 Diagnosing eclipse-induced wind changes. *Proc. R. Soc. A* 468, 1839–1850. (10.1098/rspa.2012.0007)

[15] Aplin KL, Harrison RG 2003 Meteorological effects of the eclipse of 11 August 1999 in cloudy and clear conditions. *Proc. R. Soc. Lond. A* 459, 353–371. (10.1098/rspa.2002.1042)

[16] Prenosil T 2000 The influence of the 11 August 1999 solar eclipse on the weather over Central Europe. *Meteorol. Z.* 9, 351–359.

[17] KiranKumar NVP, Purushothaman N, Santosh M 2013 Response of spectral characteristics of wind and temperature of atmospheric surface layer to the noontime annular solar eclipse. *J. Atmos. Sol.-Terr.* 97, 91–98. (10.1016/j.jastp.2013.02.017)

[18] Met Office. 2006 UK Hourly Weather Observation Data, Part of the Met Office Integrated Data Archive System (MIDAS). NCAS British Atmospheric Data Centre, 2 February 2016. See http://catalogue.ceda.ac.uk/uuid/916ac4bbc46f7685ae9a5e10451bae7c.

[19] Vaisala Road Weather Station RWS200. 2015 See http://www.vaisala.com/en/roads/products/roadweathersystems/Pages/RWS200.aspx) (accessed 21 October 2015).

[20] Davies T, Cullen MJP, Malcolm AJ, Mawson MH, Staniforth A, White AA, Wood N 2005 A new dynamical core for the Met Office’s global and regional modelling of the atmosphere. *Q. J. R. Meteorol. Soc.* 131, 1759–1782. (10.1256/qj.04.101)

[21] Tang Y, Lean HW, Bornemann J 2013 The benefits of the Met Office variable resolution NWP model for forecasting convection. *Meteorol. Appl.* 20, 417–427. (10.1002/met.1300)

[22] Harrison RG 2014 *Meteorological measurements and instrumentation*, p. 280 New York, NY: Wiley-Blackwell.

[23] Baas P, van de Wiel BJH, van den Brink L, Holtslag AAM 2012 Composite hodographs and inertial oscillations in the nocturnal boundary layer. *Q. J. R. Meteorol. Soc.* 138, 528–535. (10.1002/qj.941)

[24] Barlow JF, Halios CH, Lane SE, Wood CR 2014 Observations of urban boundary layer structure during a strong urban heat island event. *Environ. Fluid Mech.* 15, 373–398. (10.1007/s10652-014-9335-6)

[25] Blackadar AK 1957 Boundary layer wind maxima and their significance for the growth of the nocturnal inversion. *Bull. Am. Meteorol. Soc.* 38, 283–290.

[26] Thorpe AJ, Guymer TH 1977 The nocturnal jet. *Q. J. R. Meteorol. Soc.* 103, 633–653. (10.1002/qj.49710343809)

[27] Wingo SM, Knupp KR 2015 Multi-platform observations characterizing the afternoon-to-evening transition of the planetary boundary layer in Northern Alabama, USA. *Bound. Layer Meteorol.* 155, 29–53. (10.1007/s10546-014-9988-1)

[28] Girard-Ardhuin F, Bénech B, Campistron B, Dessens J, Jacoby-Koaly S 2003 Remote sensing and surface observations of the response of the atmospheric boundary layer to a solar eclipse. *Bound. Layer Meteorol.* 106, 93–115. (10.1023/A:1020837400800)

[29] Amiridis V *et al.* 2007 Aerosol Lidar observations and model calculations of the planetary boundary layer evolution over Greece, during the March 2006 total solar eclipse. *Atmos. Chem. Phys.* 7, 6181–6189. (10.5194/acp-7-6181-2007)

[30] Norton EG 2015 Vertical wind profile data from 6th November 2013 to present measured by the University of Manchester 1290 Mhz mobile wind profiler deployed on long-term observations at Met Office Research Unit, Cardington, Bedfordshire. NCAS British Atmospheric Data Centre, 11 March 2016. (http://catalogue.ceda.ac.uk/uuid/ecbecb0fbf6e4a4e93bb3ee2cf9854cd).

[31] Burt S 2016 Meteorological responses in the atmospheric boundary layer over southern England to the deep partial eclipse of 20 March 2015. *Phil. Trans. R. Soc. A* 374, 20150214 (10.1098/rsta.2015.0214) 27550762 PMC5004047

[32] Clark PA 2016 Numerical simulations of the impact of the 20 March 2015 eclipse on UK weather. *Phil. Trans. R. Soc. A* 374, 20150218 (10.1098/rsta.2015.0218) 27550770

[33] Press WH, Flannery BP, Teukolsky SA, Vetterling WT 1989 *Numerical recipes in Pascal*. Cambridge, UK: Cambridge University Press.

